# Distribution of biopsied non plaque-induced gingival lesions in a Chilean population according to the classification of periodontal diseases

**DOI:** 10.1186/s12903-018-0567-6

**Published:** 2018-06-19

**Authors:** Patricia Hernández-Ríos, Iris Espinoza, Macarena Salinas, Fernando Rodríguez-Castro, Mauricio Baeza, Marcela Hernández

**Affiliations:** 1 Department of Conservative Dentistry, Faculty of Dentistry, University of Chile, Avenida Sergio Livingstone 943, Comuna de Independencia, Santiago, Chile; 2 Department of Pathology, Faculty of Dentistry, University of Chile, Sergio Livingstone 943, Independencia, Santiago, Chile; 3 Faculty of Dentistry, University of Chile, Sergio Livingstone 943, Independencia, Santiago, Chile; 4 Dentistry Unit, Faculty of Health Sciences, Universidad Autónoma de Chile, Pedro de Valdivia, 425 Santiago, Chile

**Keywords:** Non plaque-induced gingival lesions, Classification of periodontal diseases

## Abstract

**Background:**

Many gingival lesions are not induced by plaque. The aim of this study was to analyze the frequency of biopsied non-plaque-induced gingival lesions (NPIGL) in a Chilean population.

**Methods:**

One thousand twelve cases of biopsied gingival lesions with confirmed anatomopathologic diagnosis were included, from the records of the Oral Pathology Referral Institute (OPRI), Faculty of Dentistry, University of Chile, between years 1990 and 2009.

**Results:**

The most frequent non plaque-induced gingival lesions categories from biopsied cases included hyperplastic lesions, malignancies and benign neoplasms. The most frequent diagnoses in each category were fibrous hyperplasia (35.47%), squamous cell carcinoma (3.85%) and giant cell fibroma (2.08%), respectively. From all lesions, only 8.3% fitted in the specified categories of the current classification of periodontal diseases.

**Conclusions:**

The most frequent biopsied NPIGL were hyperplastic lesions and neoplasms. These categories represent relevant lesions to be included in a future periodontal classification system to improve the care needs of the patients, as well as early diagnosis and treatment.

## Background

Gingival health is under the clinical scope of the periodontist. The vast majority of pathologies affecting the gingiva (gingivitis and periodontitis) are induced by dental plaque [1]; however, gums are also a frequent site for conditions and lesions that obey to a wide range of etiologies different from bacterial biofilm, showing a diversity of clinical features that can sometimes overlap with gingivitis and periodontitis [2].

Non-plaque-induced gingival lesions (NPIGL) encompass a group of pathologies that are not primarily caused by plaque and usually do not disappear after plaque removal, even when the severity of their clinical manifestations depends on the interaction with the underlying bacterial plaque [3]. This section, as part of the prevailing classification system for periodontal diseases and conditions established by the American Academy of Periodontology (AAP) in 1999 [4], includes a wide range of disorders that affect the gingiva, including gingival diseases of specific bacterial, viral and fungal origin; genetic origin; gingival manifestations of systemic conditions; traumatic lesions; foreign body reactions and not otherwise specified.

Nevertheless, the gingiva seems to be a common target tissue for other non-neoplastic and neoplastic lesions, and its close relationship with the periodontal ligament might give rise to unique lesions [5–7]. The evidence shows that an important proportion of oral mucosal lesions, different from those involving the current classification of periodontal diseases, are located in the gingiva among the most affected sites [5, 8], accounting for 19 to 50% of all lesions in oral mucosa. Some of them, such as peripheral giant cell granuloma and cemento-ossifying fibroma, have been proposed to be specific of gingiva [9]. Even though these pathologies may involve specific, invasive or even life-threatening entities, periodontists might not be aware of them, despite being in a key position to contribute to their early diagnosis [1].

At present, a limited number of epidemiological studies assess the prevalence of NPIGL [1, 2, 6, 10] and none of them considers the current classification of periodontal diseases. The aim of this study was to analyze the frequency of NPIGL in the Chilean population registered in the Oral Pathology Referral Institute (OPRI), Faculty of Dentistry, University of Chile, between years 1990–2009.

## Methods

The study sample consisted of biopsy specimens submitted to the Oral Pathology Referral Institute (OPRI), Faculty of Dentistry, University of Chile, between 1990 and 2009, referred from public and private healthcare centers and institutions. The cases were included if they affected the gingiva and had their definite anatomopathologic diagnosis confirmation. Uncertain diagnoses were re-examined and confirmed by a trained oral pathologist (IE). Exclusion criteria were salivary gland lesions (mucocele, pleomorphic adenoma, polymorphous low-grade adenocarcinoma) that may have reached the gingival area by extension, externalized intraosseous lesions (osteomyelitis, maxillary cysts and odontogenic tumors) and nonspecific inflammatory processes that could not be distinguished from gingival plaque-induced gingivitis or periodontitis (nonspecific inflammation, nonspecific ulcer and abscesses). This study was independently reviewed and approved by the Ethics Committee of the Faculty of Dentistry, University of Chile, and was conducted in accordance with the Helsinki Declaration of 1975, as revised in 2000.

Data regarding demographic information, including age, gender and location, and the histopathological diagnoses were collected from the clinical and anatomopathologic records, respectively. A total of 1.012 NPIGL were finally included. The data were analyzed with Stata software, version 12 (STATA Corp., Texas, USA) and Microsoft Excel software. The results were expressed as total and relative frequencies.

## Results

The total number of cases and the frequency for each respective histopathological diagnosis are described in Table 1. The most frequent NPIGL from total cases (*n* = 1012) included types of reactive hyperplastic lesions, mainly fibrous hyperplasia (*n* = 359, 35.47%), pyogenic granuloma (*n* = 190, 18.77%), peripheral giant cell granuloma (*n* = 98, 9.68%), peripheral cemento-ossifying fibroma (*n* = 58, 5.73%) and epithelial hyperplasia and/or hyperkeratosis (*n* = 50, 4.94%). The sixth most frequent diagnosis and the most common type of cancer was squamous cell carcinoma (*n* = 39, 3.85%), followed by other kind of lesions, including amalgam tattoo (*n* = 24, 2.37%), papilloma (*n* = 22, 2.17%), giant cell fibroma (the most frequent benign neoplasm; *n* = 21, 2.08%); and intraepithelial dysplasia (*n* = 18, 1.78%). Other relevant malignancies found in this study were non-Hodgkin lymphoma (*n* = 14, 1.38%), Kaposi’s sarcoma (*n* = 4, 0.4%) and melanoma (*n* = 3, 0.3%).

**Table 1:** Frequency of NPIGL according to histopathological diagnosis

Histopathological diagnosis	n (%)
Fibrous Hyperplasia	359 (35.47)
Pyogenic Granuloma	190 (18.77)
Peripheral Giant Cell Granuloma	98 (9.68)
Peripheral Cemento-Ossifying Fibroma	58 (5.73)
Epithelial Hyperplasia/Hyperkeratosis	50 (4.94)
Squamous Cell Carcinoma	39 (3.85)
Amalgam Tatoo	24 (2.37)
Papilloma	22 (2.17)
Giant Cell Fibroma	21 (2.08)
Intraepithelial Dysplasia	18 (1.78)
Hyper-melanogenesis/Oral Melanotic Macule	16 (1.58)
Benign Mucosal Pemphigoid	15 (1.48)
Non-Hodgkin Lymphoma	14 (1.38)
Haemangioma	11 (1.09)
Nevus	6 (0.59)
Hereditary Gingival Fibromatosis	6 (0.59)
Gingival Cyst of the Adult	5 (0.49)
Odontogenic Fibroma	5 (0.49)
Soft Tissue Metastases (primary not specified)	5 (0.49)
Lipoma/ Fibrolipoma	5 (0.49)
Kaposi’s sarcoma	4 (0.40)
Lichen Planus	4 (0.40)
Melanoma	3 (0.30)
Neurofibroma	3 (0.30)
Haematoma	3 (0.30)
Foreign Body Reaction	3 (0.30)
Intraoral Herpes	3 (0.30)
Fibrosarcoma	3 (0.30)
Tuberculosis	2 (0.20)
Pemphigus Vulgaris	2 (0.20)
Oral Focal Mucinosis	2 (0.20)
Peripheral Ameloblastoma	2 (0.20)
Benign Spindle Cell Tumor	2 (0.20)
Dilantin-induced Gingival Hyperplasia	1 (0.10)
Traumatic Neuroma	1 (0.10)
Verruciform Xanthoma	1 (0.10)
Spindle Cell Carcinoma	1 (0.10)
Allergic Stomatitis	1 (0.10)
Congenital Epulis of the Newborn	1 (0.10)
Malignant Fibrohystiocitoma	1 (0.10)
Leukemia	1 (0.10)
Varicosity	1 (0.10)
Total	1012 (100.00)

The diagnoses were clustered into nine categories, according to its pathological nature, as shown in Table 2. The reactive hyperplastic lesions category was the most numerous by far, reaching 76.28% of all the lesions (*n* = 772). The second and third most common categories were “malignant neoplasms” (*n* = 71, 7.02%) and “benign neoplasms and other benign tumors” (*n* = 65, 6.42%), which included odontogenic cysts and tumors, benign tumors with unspecific etiology and vascular malformations. They were followed by “infectious lesions” and “foreign body reactions and exogenous inclusion”, which showed the same frequency of cases (*n* = 27, 2.67%), while “potentially malignant disorders” and “mucocutaneous disorders” were 2.17% (*n* = 22) and 1.78% (*n* = 18) of all biopsies, respectively. The least frequent groups of lesions found in this study were “genetic lesions” (*n* = 6, 0.59%) and “others” (*n* = 4, 0.4%), that included the diagnoses of haematoma and a varicosity.

**Table 2:** Classification of NPIGL according to pathological nature

Pathological nature	Histopathological diagnosis	Total n (%)
Reactive hyperplastic lesions	Fibrous Hyperplasia, Pyogenic Granuloma, Peripheral Giant Cell Granuloma, Peripheral Cemento-Ossifying Fibroma, Hyper-melanogenesis/Oral melanotic macule, Epithelial Hyperplasia/Hyperkeratosis, Dilantin-induced Gingival Hyperplasia, Traumatic Neuroma.	772 (76.28)
Malignant neoplasms	Squamous Cell Carcinoma, Non-Hodgkin Lymphoma, Soft –tissue Metastases, Melanoma, Kaposi’s sarcoma, Fibrosarcoma, Spindle Cell Carcinoma, Malignant Fibrohystiocitoma, Leukemia.	71 (7.02)
Benign neoplasms and other benign tumors	Giant Cell Fibroma, Haemangioma, Nevus, Gingival Cyst of the Adult, Odontogenic Fibroma, Lipoma/ Fibrolipoma, Neurofibroma, Oral Focal Mucinosis, Verruciform Xanthoma, Benign Spindle Cell Tumor, Congenital Epulis of the newborn, Peripheral Ameloblastoma.	65 (6.42)
Infectious lesions	Papilloma, Intraoral Herpes, Tuberculosis	27 (2.67)
Foreign body reaction and exogenous inclusion	Amalgam Tatoo, Foreign Body Reaction	27 (2.67)
Potentially malignant disorders	Precancerous lesion: Intraepithelial Dysplasia Precancerous condition: Lichen Planus	22 (2.17)
Mucocutaneous disorders	Benign Mucosal Pemphigoid, Pemphigus Vulgaris, Allergic Stomatitis	18 (1.78)
Genetic lesions	Hereditary Gingival Fibromatosis	6 (0.59)
Others	Haematoma, Varicosity	4 (0.40)
Total		1012 (100.00)

As shown in Table 3, 65.02% (*n* = 658) of total cases were females and 34.98% (*n* = 354) were males. The category “reactive hyperplastic lesions” constituted the most common category for both genders and slightly prevailed in females (*n* = 524, 67.88%). Followed by “malignant neoplasms” (*n* = 38, 53.52% in females), “benign neoplasms and other benign tumors” (*n* = 39, 60.0% in females). “mucocutaneous disorders”, “foreign body reactions and exogenous inclusion” and “infectious lesions” also exhibited a higher number of cases in females than in males, while “potentially malignant disorders” and “others”, were equally distributed between genders. Conversely, genetic lesions were more common in men than in women (83.33% versus 16.67%).

**Table 3:** Distribution of NPIGL categorized by pathological nature according to gender, location and age

Pathological Nature	Gender		Location			Age								
	Female n (%)	Male n (%)	Maxilla n (%)	Mandible n (%)	Non Reported n (%)	0–9 n (%)	10–19 n (%)	20–29 n (%)	30–39 n (%)	40–49 n (%)	50–59 n (%)	60–69 n (%)	≥70 n (%)	Non Reported n (%)
Reactive hyperplastic lesions	524 (67.88)	248 (32.12)	375 (48.58)	366 (47.41)	31 (4.02)	39 (5.05)	75 (9.71)	99 (12.82)	122 (15.08)	114 (14.76)	145 (18.78)	115 (14.89)	58 (7.51)	5 (0.64)
Malignant neoplasms	38 (53.52)	33 (46.48)	38 (53.52)	29 (40.85)	4 (5.63)	1 (1.40)	3 (4.22)	5 (7.04)	8 (11.26)	4 (5.63)	14 (19.71)	18 (25.35)	18 (25.35)	0 (0.00)
Benign neoplasms and other benign tumors	39 (60.00)	26 (40.00)	27 (41.54)	36 (55.38)	2 (3.08)	11 (16.92)	11 (16.92)	6 (9.23)	11 (16.92)	10 (15.38)	8 (12.30)	6 (9.23)	2 (3.07)	0 (0.00)
Infectious lesions	14 (51.85)	13 (48.15)	16 (59.26)	9 (33.33)	2 (7.41)	3 (11.11)	5 (18.51)	1 (3.70)	10 (37.03)	1 (3.70)	2 (7.40)	3 (11.11)	2 (7.40)	0 (0.00)
Foreign Body-reaction and exogenous inclusion	16 (59.26)	11 (40.74)	11 (0.74)	14 (51.85)	2 (7.41)	0 (0.00	3 (11.11)	3 (11.11)	2 (7.40)	7 (25.92)	4 (14.81)	4 (14.81)	4 (14.81)	0 (0.00)
Potentially malignant disorders	11 (50.00)	11 (50.00)	12 (54.55)	7 (31.82)	3 (13.64)	0 (0.00)	0 (0.00)	1 (4.54)	1 (4.54)	5 (22.72)	4 (18.18)	7 (31.81)	4 (18.18)	0 (0.00)
Mucocutaneous disorders	13 (72.22)	5 (27.78)	9 (50.00)	7 (38.89)	2 (11.11)	0 (0.0)	0 (0.0)	2 (11.11)	1 (5.55)	2 (11.11)	0 (0.00)	7 (38.88)	6 (33.33)	0 (0.00)
Genetic lesions	1 (16.67)	5 (83.33)	1 (16.67)	2 (33.33)	3 (50.00)	2 (33.33)	2 (33.33)	0 (0.00)	0 (0.00)	1 (16.66)	1 (16.66)	0 (0.00)	0 (0.00)	0 (0.00)
Others	2 (50.00)	2 (50.00)	1 (25.00)	3 (75.00)	0 (0.00)	0 (0.00)	0 (0.00)	0 (0.00)	1 (25.00)	0 (0.00)	1 (25.00)	2 (50.00)	0 (0.00)	0 (0.00)
Total	658 (65.02)	354 (34.98)	490 (48.42)	473 (46.74)	49 (4.80)	56 (5.53)	99 (9.78)	117 (11.56)	156 (15.41)	144 (14.22)	179 (17.68)	162 (16.00)	94 (9.28)	5 (0.49)

According to their location (Table 3), a slight majority of cases were obtained from the maxillary gingiva (*n* = 490, 48.42%), in comparison with the mandible (*n* = 473, 46.74%). “Reactive hyperplastic lesions” and “malignant neoplasms” categories were mildly more frequent in maxilla (*n* = 375, 48.58%; and *n* = 38, 53.52%, respectively), while “benign neoplasms and other benign tumors” were mainly located in the mandible (*n* = 36, 55.38%). It must be considered that there were a total of 49 cases (4.80%) in which the location of the lesion was not reported.

NPIGL were diagnosed in patients with a wide range of ages, from 0 to 86 years, with a mean age of 43.3 years. The occurrence of NPIGL increased progressively through aging, reaching peaks in the sixth (*n* = 179) and seventh decades (*n* = 162) (Table 3). The age was not reported in 5 cases, and all of them corresponded to reactive hyperplastic lesions. The majority of reactive hyperplastic lesions were diagnosed between 50 and 59 years of age (*n* = 145), corresponding to 18.78%. Malignant neoplasms were strikingly more frequent in patients from 50 years and older, while benign neoplasms and other benign tumors were scattered mainly from the first to the seventh decade of life.

NPIGL found in this study were also classified according to the corresponding seven specified NPIGL categories reported by the 1999 AAP Classification to assess their frequencies (Table 4). These categories included lesions of specific bacterial, viral or fungal origin (1–3); lesions of specific genetic origin (4); gingival manifestations of systemic conditions (5); traumatic lesions (6) and foreign body reactions (7). Only 8.01% of the lesions found in the current study fitted in the specified categories of the 1999 classification of periodontal diseases and conditions. However, the most prevalent categories found in the present study, namely reactive hyperplastic lesions, malignant neoplasms, benign neoplasms and other benign tumors and potentially malignant disorders matched to the “not otherwise specified” category of the AAP classification.

**Table 4:** Frequency of Biopsied Non-Plaque Induced Gingival Lesions according to AAP Classification

AAP	Current category	Frequency n (%)
(1–3) Specific bacterial, viral or fungal origin.	Infectious lesions	27 (2.67)
(4) Genetic origin	Genetic lesions	6 (0.59)
(5) Gingival manifestations of systemic conditions	Mucocutaneous disorders	18 (1.78)
(6) Traumatic lesions	Others (Haematoma)	3 (0.29)
(7) Foreign body reactions	Foreign body reactions and exogenous inclusion	27 (2.67)
Not otherwise specified	Reactive hyperplastic lesions, malignant neoplasms, benign neoplasms and other benign tumours, potentially malignant lesions, others (varicosity).	931 (91.99)
	Total	1012 (100.00)

## Discussion

Even if bacterial biofilm constitutes the main etiological factor for the most prevalent diseases that affect the periodontal tissues, the gingiva is also affected by different types of lesions, which are not primarily caused by bacterial plaque. Furthermore, some of them can reach considerable prevalence and/or high pathological relevance, up to jeopardize the life of an individual. We performed a retrospective study to analyze the frequency of non-plaque-induced gingival lesions in a Chilean population and we also compared and adjusted the results according to the NPIGL categories established in the current classification of periodontal diseases and conditions [4].

NPIGL found in this study were grouped according to nine categories. From the most to the least prevalent one, they corresponded to reactive hyperplastic lesions; malignant neoplasms; benign neoplasms and other benign tumors; infectious lesions; foreign body reactions and exogenous inclusions; mucocutaneous disorders; potentially malignant disorders; genetic lesions; and others. In accordance with previous reports [1, 2, 6–8], the majority of the lesions in gingiva and in the oral cavity were non neoplastic, and mainly hyperplastic-reactive in nature (76.28%).

Among the reactive hyperplastic lesions, the most frequent diagnosis was fibrous hyperplasia (35.47%), followed by pyogenic granuloma (18.77%). Peripheral giant cell granuloma, peripheral cemento-ossifying fibroma and epithelial hyperplasia reached also high frequencies. Peripheral giant cell granuloma and peripheral cemento-ossifying fibroma have a special relevance in the pathology of periodontal tissues, considering not only their frequency, but also their particular genesis, because both of these lesions have been ascribed to the periodontal ligament and are exclusively present in the gingiva [9, 11, 12].

The former category was followed in frequency by malignant (7.02%) and benign neoplasms (6.42%), which is generally in accordance with previous reports. Although some studies found a higher prevalence of benign neoplasms over malignancies, they included other non-neoplastic tumor-like reactive lesions, such as ossifying peripheral fibroma, which contributed to increase their prevalence [1, 7]. More strikingly, the prevalence of gingival cancer was similar to other reports with frequencies ranging from 6.5 to 8% of total gingival lesions [2, 6, 7, 13]. While reactive hyperplastic lesions reached peak prevalence levels between 50 and 59 years, it is interesting to notice that malignant neoplasms were highly represented at older ages, while benign neoplasms were almost evenly distributed.

According to previous studies [2, 6, 7, 13], the most common type of cancer in this work was squamous cell carcinoma (SCC), which reached a frequency of 3.85% of all lesions, representing the sixth most frequent diagnosis after cases of reactive hyperplastic lesions. This highlights the relevance of SCC and malignancies early detection and diagnosis associated with periodontal tissues examination.

Moreover, the category potentially malignant disorders (PMDs) was considered, according to the World Health Organization (WHO), as the risk of malignancy of a lesion or condition during its initial diagnosis or in a future time. The group can be divided into two categories, namely precancerous lesion, a benign condition with histological alterations that has an increased risk of malignant conversion; and precancerous condition, a disease or habit that relates to a raised risk of precancerous lesion or cancer initiation, without the necessity of clinical alterations [14]. In this work, intraepithelial dysplasia (precancerous lesion) and lichen planus (precancerous condition) were both included into PMDs category, that reached a frequency of 2.17%, slightly lower than previously reported [8].

Gingival pathologies were introduced to the classification of periodontal diseases and conditions for the first time in 1999, as a new section with a detailed classification of gingival diseases and lesions, and were subcategorized into dental plaque-induced diseases and lesions that were not primarily associated with dental plaque (NPIGL). In this study, biopsied NPIGL were distributed according to 1999 non-plaque-induced gingival lesion subcategories [4]. However, the vast majority of NPIGL reported (91.99%), including the most common groups (reactive hyperplastic lesions, malignant neoplasms and benign neoplasms and tumors), did not fit into the specified categories. However, the most prevalent NPIGL found in this and previous reports, namely reactive hyperplastic lesions, origin as a response to local irritation and/or chronic trauma from calculus, restorations and iatrogenic factors [9]. From this point of view, reactive hyperplastic lesions might fit in the AAP category of traumatic lesions to physical injury, bearing in mind that they are not derived from acute, but chronic low-trauma.

The present study included pyogenic granuloma among NPIGL, a reactive lesion that may be caused by persistent local irritation or trauma. This lesion has been categorized into the 1999 classification of periodontal diseases and conditions as a part of “pregnancy associated dental plaque-induced gingival diseases”. From a clinical point of view, this lesion corresponds to granuloma gravidarum [4], a pyogenic granuloma that is modified by hormonal changes associated with pregnancy. Although pyogenic granuloma can be modified by oral hygiene and hormones, it represents a reactive hyperplastic response that is not necessarily caused by gingival plaque and do not disappear after plaque removal and in consequence, it is not primarily associated with dental plaque [11, 15].

Bearing in mind that classification systems should aid clinicians in early diagnosis and health care organization, it would be recommendable to specify more categories of relevant gingival lesions in future periodontal classification systems, based on their high prevalence (reactive hyperplastic lesions), gingival tissue specificity (peripheral giant cell granuloma and peripheral cement-ossifying fibroma), early diagnosis (potentially malignant disorders) and/or high morbidity and mortality (squamous cell carcinoma).

The performance of a retrospective study implicates some limitations and sources of bias, particularly, the lack of complete clinical and demographic information (such as age and location in some of the cases). Additionally, it is important to bear that the present study only included lesions subjected to biopsy and consequently, those cases in which biopsy is not primarily indicated, such as in some viral, fungal, traumatic or other nonspecific inflammatory lesions, might be underestimated. Even though clinical history along with histologic studies usually guides to a definite diagnosis, in the case of some complex entities such as certain immune diseases and spindle cell tumors, other complementary exams may be necessary. All in all, to the best of our knowledge, this study represents the first report of the frequency of non-plaque-induced gingival lesions in a Chilean population and the first study to compare the prevalence of NPIGL with 1999 periodontal disease classification.

## Conclusions

The most frequent biopsied NPIGL were hyperplastic lesions and neoplasms. Based on their high prevalence, relevant morbidity, mortality, and/or gingival specificity, the current study highlights the importance to evaluate the inclusion of new NPIGL categories and prevalent subtypes of reactive hyperplastic lesions, benign neoplasms and malignancies in future periodontal classification systems. This might contribute to organize patients’ care needs as well as early diagnosis and well-timed treatment.

## Abbreviations

AAP: American Academy of Periodontology
NPIGL: Non-Plaque-Induced Gingival Lesions
OPRI: Oral Pathology Referral Institute
PMDs: Potentially Malignant Disorders
WHO: World Health Organization
